# Magnetic Fluids: The Interaction between the Microstructure, Macroscopic Properties, and Dynamics under Different Combinations of External Influences

**DOI:** 10.3390/nano14020222

**Published:** 2024-01-19

**Authors:** Petr Ryapolov, Anastasia Vasilyeva, Dariya Kalyuzhnaya, Alexander Churaev, Evgeniy Sokolov, Elena Shel’deshova

**Affiliations:** Department of Nanotechnology, Microelectronics, General and Applied Physics, Faculty of Natural Sciences, Southwest State University, 50 Let Oktyabrya Street, 94, 305040 Kursk, Russia; vasilyeva.ao@mail.ru (A.V.); kalyuzhnaya.dariya@yandex.ru (D.K.); tchyalex@yandex.ru (A.C.); evgeniysokolov1@yandex.ru (E.S.); blackberry__@mail.ru (E.S.)

**Keywords:** magnetic fluids, smart materials, magnetic field, magnetoviscous effect, microfluidics

## Abstract

Magnetic fluids were historically the first active nano-dispersion material. Despite over half a century of research, interest in these nano-objects continues to grow every year. This is due to the impressive development of nanotechnology, the synthesis of nanoscale structures, and surface-active systems. The unique combination of fluidity and magnetic response allows magnetic fluids to be used in engineering devices and biomedical applications. In this review, experimental results and fundamental theoretical approaches are systematized to predict the micro- and macroscopic behavior of magnetic fluid systems under different external influences. The article serves as working material for both experienced scientists in the field of magnetic fluids and novice specialists who are just beginning to investigate this topic.

## 1. Introduction

The field of magnetic soft materials represents a burgeoning interdisciplinary scientific discipline that has emerged in recent decades. It encompasses tasks primarily associated with condensed matter physics, magnetism, magnetic hydrodynamics, inorganic and organic chemistry, colloid chemistry, computational and computer modeling, acoustics, engineering, and applied sciences. Magnetically soft materials encompass magnetic fluids, magnetic elastomers, and magnetic gels containing magnetic nanoparticles, all susceptible to modification via various magnetic and non-magnetic inclusions.

Historically, magnetic fluids (MFs) took the lead, constituting a colloidal system of magnetic material-coated nanoparticles dispersed in a carrier liquid. Magnetic fluid harbors single-domain superparamagnetic nanoparticles, typically around 10 nm in size. These early materials are considered the pioneers of ‘smart’ nano-dispersed materials [1,2], which are extensively explored in the scientific literature [3,4,5] and applied across various devices and technologies [6,7,8,9]. Magnetic fluid has a unique combination of fluidity and the ability to respond to an external magnetic field and has found an application in seals [7], controlled shock absorbers [6], various sensors [8], and acoustic systems [9]. The remarkable progress in nanotechnology and the ability to synthesize materials and structures over the past few decades has enabled the conceptualization of magnetic fluids as multiphase systems. In this context, magnetic nanoparticles function as distinct elements with a controlled structure that can undergo surface changes facilitated by specific surfactants, interacting selectively with certain biological entities or organic compounds [10].

Although magneto-fluidic systems with diverse compositions find application in technology and medicine, their macroscopic properties, microstructure, and behavioral dynamics are significantly influenced by external factors. Accurately predicting the micro- and macroscopic responses of magneto-fluidic systems, which is pivotal for developing smart materials under external influences, represents a crucial scientific challenge. Consequently, studying the interaction between the microstructure and macroscopic properties of magneto-fluidic systems becomes imperative. This involves examining how external non-invasive magnetic, mechanical, and acoustic influences collectively impact these systems, alongside exploring the dynamics of the non-magnetic inclusions within these systems when subjected to external inhomogeneous magnetic fields.

## 2. Nano-Disperse Magnetic Fluids: Discovery and Research Interest

Article [11] extensively details the historical synthesis process of stable colloids from magnetic nanoparticles, typically with an effective diameter of ~10 nm or larger. These colloids were initially termed magnetic fluids [11], with their dynamics termed ferrohydrodynamics [12]. Magnetic fluids represent unique artificially created materials. Magnetostatic bacteria are capable of detecting magnetic nanoparticles (MNPs) [13], yet stable liquid systems exhibiting ferromagnetic properties are absent. In the early 19th century, eminent physicists Michael Faraday and Thomas J. Seebeck studied magnetic dust under external magnetic field influences [14]. The system they investigated was unstable and settled quickly. Subsequently, Elmore measured the magnetization curves of micro-sized particles dispersed in a carrier liquid [15]. Magnetic fluids, as studied today, were first synthesized about 60 years ago in the USA [1]. They stood as pioneering artificial nano-dispersed material and became subjects of scientific research [2]. Magnetic fluids have found application in various technical devices [2,16,17], even predating the term ‘nanotechnology’ [18].

**Figure 1:**
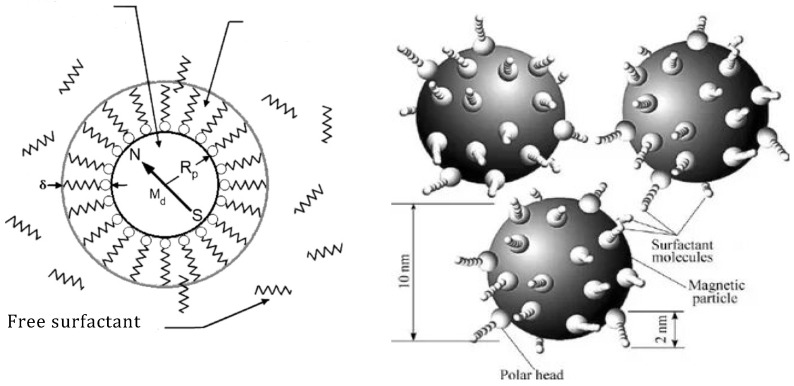
A schematic representation of the MNP in the magnetic fluid coated with a surfactant. Adapted with permission from Ref. [20]. 1998, Odenbach, S.

A nano-disperse magnetic fluid constitutes a colloidal system of MNPs (magnetic nanoparticles) ranging from 5 to 20 nm in diameter. These MNPs are coated with a stabilizing shell—a surfactant—dispersed within a liquid carrier. Figure 1 [19,20,21,22] provides a schematic representation of such a particle. The properties of the magnetic fluid are significantly influenced by the size of the MNPs, their concentration, and the types of surfactants used.

Magnetic particles within the magnetic fluid exhibit monodomain characteristics and rely on magnetic dipoles with a constant magnetic moment for description. Simultaneously, their small size prevents sedimentation at room temperature, which is facilitated by Brownian fluctuations inducing random movements of MNPs. Consequently, they uniformly disperse throughout the entire volume of the liquid carrier.

The most important characteristic of magneto-fluidic systems lies in their combination of fluidity and their capability to react (through changes in physical properties and microstructure and dynamics of the interface) to external magnetic, mechanical, and acoustic influences. The unique capabilities of magnetic fluid classify them as ‘smart’ materials and generate widespread interest among physicists, material scientists, and engineers. Ronald Rosensweig’s introduction to fundamental equations for the hydrodynamics of magnetic forces in a quasi-homogeneous magnetizable liquid medium laid the groundwork for creating a new scientific direction: ‘ferrohydrodynamics’ [2,23,24]. This field described phenomena such as thermomagnetic convection, surface instability, the levitation of a permanent magnet, and other phenomena. Subsequent experimental studies established the dependence of magnetic fluid viscosity on the external magnetic field (the magnetoviscous effect) [2] and experimentally revealed a rotational effect [12]. These experiments’ results could only be explained by considering the influence of relaxation processes. Study [2] proposes a physical model that considers the internal rotation of magnetic nanoparticles. However, this model does not account for the fact that the rotational effect occurs only in inhomogeneous magnetic fields, as demonstrated in experiment [25], and explaining this effect requires considering the structuring of magnetic fluid in an inhomogeneous magnetic field.

Engineers and scientists have shown interest in magnetic fluids. Many scientific works systematically organizing the structure, dynamics, and physical properties of magnetic fluid have been published in classical monographs [2,9] and review articles [22,26,27,28,29,30,31,32,33].

**Figure 2:**
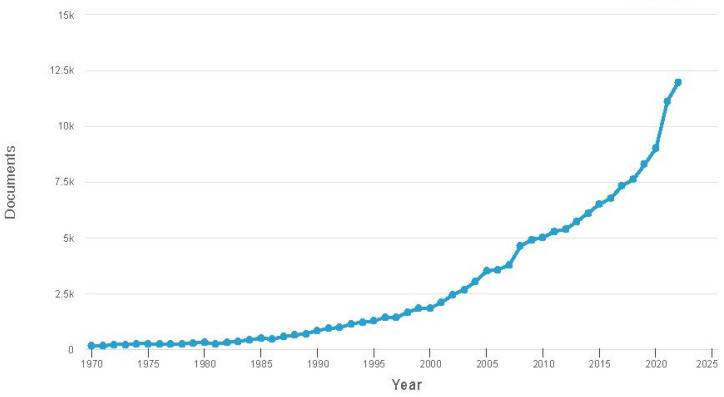
Dynamics of the number of publications devoted to magneto-fluidic systems, according to the Scopus database.

Nanotechnology has been evolving over the past 15 years, sparking renewed interest in magnetic fluids. Researchers have started viewing magnetic fluid from a different perspective. Currently, magnetic fluid is recognized as a multiphase system where magnetic nanoparticles exist as distinct elements with controlled structures and properties. Heightened interest has led to a significant surge in publications on this topic, which is reflected in Figure 2.

Magnetic fluid and magnetic nanoparticles find numerous applications, particularly in the field of biomedicine [34,35,36]. These particles serve as contrast agents in magnetic resonance imaging [37,38]. Researchers are actively investigating interactions between particles, the formation of chain aggregates and flexible clusters, and the impact of the microstructure on the macroscopic properties of magnetic fluid through both experimental and theoretical studies [39,40]. The development of magneto-fluidic systems has made it possible to significantly alter viscosity under the influence of an external magnetic field, showcasing a giant magnetoviscous effect.

The capabilities of modern chemistry enable the synthesis of various magnetic composite anisotropic MNPs achieved through extensive efforts in refining production methods that control the material, size, shape, structure, and modification of the MNP surface [41,42]. Functionalizing the MNP surface allows their utilization in creating biosensors [43], targeted drug delivery [44], magnetic separation, and the concentration of various materials [45,46], including biological objects. For instance, the antibodies of various viruses are employed in diagnostics [47], such as COVID-19 [48]. Coupled with a high-frequency magnetic field, they have the ability to generate heat and induce magnetic hyperthermia [49,50,51] for cancer treatment.

Another prevalent aspect within magneto-fluidic systems is microfluidic self-assembly [52,53]. This direction allows for the creation of distinctive nano- and microstructures when subjected to various external factors, encompassing magnetic objects [54], as well as non-magnetic objects within the magnetic fluid [55].

Presently, the exploration of magneto-fluidic systems constitutes a multidisciplinary field, encompassing condensed matter physics, magnetism, hydrodynamics, inorganic and organic chemistry, colloidal chemistry, computational and computer modeling, acoustics, engineering, and applied sciences.

## 3. General Information about Magnetic Fluids

**Figure 3:**
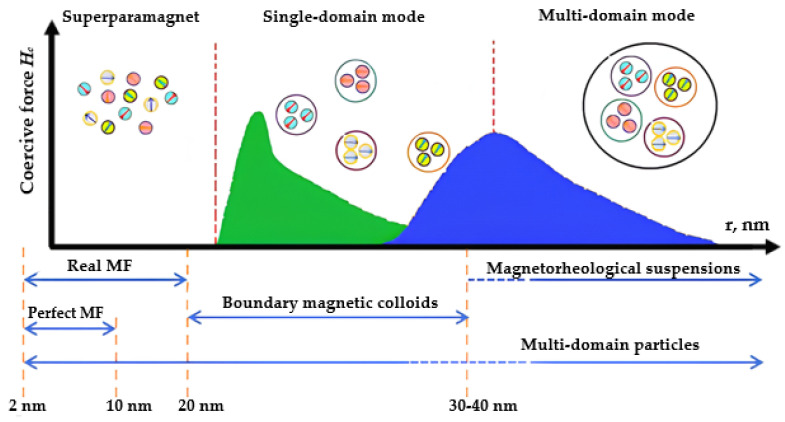
Influence of MNPs size on its magnetic structure and coercive force. Adapted with permission from Ref. [57]. 2010, Faraji, M.; Yamini, Y.; Rezaee, M.

Magnetic fluid is a multiphase system including solid MNPs, a surfactant stabilizer that prevents the aggregation of particles, and a carrier liquid [56], as previously mentioned. Solid fillers in the form of MNPs, typically ranging from 5 to 20 nm in size, are most frequently utilized (Figure 3) and derived from ferro- or ferrimagnetic metals and metal oxides. Figure 3 schematically depicts the dependence of a coercive force on MNP size [23,57,58]. Reducing the particle size of a magnetic material, inherently multi-domain, results in the formation of superparamagnetic single-domain particles [59,60]. This paper specifically focuses on MNPs based on an Fe_3_O_4_ magnetite. Fe_3_O_4_ magnetite possesses a reversed spinel-type structure, and its axis of light magnetization corresponds to the diagonal of the cube (third-order axis). Table 1 provides the physical parameters of magnetite.

**Figure 4:**
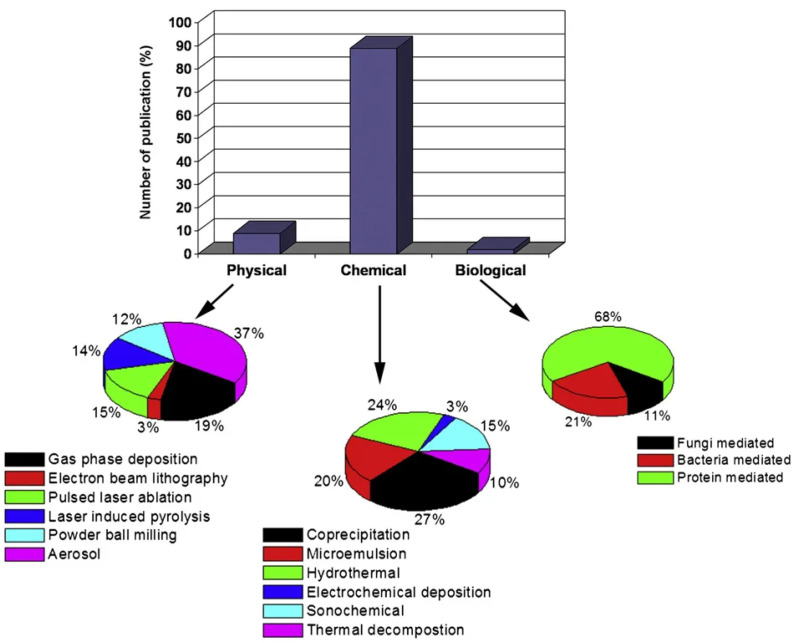
Publications on the technology of magnetic fluid synthesis. Adapted with permission from Ref. [22]. 2015, Novopashin, S.A.; Serebryakova, M.A.; Khmel, S.Y.

One can obtain superparamagnetic MNPs through physical, chemical, and biological methods. The distribution of work on the technology of magnetic fluid synthesis is depicted in Figure 4, as presented in the review [22].

Numerous works focus on chemical methods, with top-down methods being the first ones employed historically. This method involves the prolonged grinding of magnetite particles in ball mills. Subsequently, magnetite particles underwent crushing through ultrasonic treatment [63,64,65,66]. The widespread use of magnetic fluid can be attributed primarily to the advancement of chemical synthesis and the development of bottom-up technologies.

The most widespread method for synthesizing magnetic fluid is based on chemical condensation. This technology involves depositing low-frequency magnetite using a concentrated alkali solution from an aqueous solution of salts of ferrous and trivalent iron. When heated, the colloidal precipitate of MNPs is mixed with surfactants. The colloidal precipitate undergoes dissolution in the carrier liquid, a process termed peptization. The chemical condensation method is simple and technologically efficient, allowing the production of magnetic fluid based on various carrier liquids. It stands as the primary method in modern technology for magnetic fluid production [12].

The varieties of magnetic fluid distinguish themselves depending on the type of stabilization in the colloidal system. Magnetic fluid types are categorized as sterically stabilized. In this case, surfactant molecules form a solvate shell around the MNPs. Surfactant molecules physically or chemically bond with a particle. Figure 1 illustrates the model of such a particle. The surfactant molecule acts like a spring, with one end attached to the MNPs. When the particles approach, surfactant molecules contract, repelling the particles and preventing coagulation.

Another type of stabilization is ion stabilization, as proposed by Massart [67,68]. Electrostatic repulsion ensures the chemical and physical stability of the ionic magnetic fluid. The effectiveness of electrostatic repulsion strongly depends on the acidity and type of carrier fluid, which includes non-magnetic liquids such as kerosene, liquid hydrocarbons, oils, water, paraffin, organosilicon, and others. Selecting the appropriate surfactant is necessary for each type of carrier fluid. Ionic (electrostatic) stabilization is practical, mainly in the case of water-based magnetic fluids, while steric/steric + electrostatic stabilization proves useful for a wide variety of non-polar and polar carriers.

The chemical condensation method appears simple, but even a slight modification in the technological process can alter the structure of MNPs and the physical properties of the magnetic fluid. Monitoring MNPs’ structure and parameters constitutes one of the most crucial tasks. Researchers use this control to enhance the technology for producing magnetic fluid. Resolving this issue necessitates employing the following various physical methods: directly observing MNPs through atomic force microscopy, magnetic force microscopy, transmission electron microscopy, scanning electron microscopy, small-angle X-ray scattering, and magnetogranulometric analysis. Additionally, non-invasive methods, which rely on mechanical and acoustic influences and do not necessitate optical transparency of the medium, are also utilized. These noninvasive methods enable the study of dispersed magnetic fluid without replication.

## 4. Physical Properties and Structure of Magnetic Fluids

The magnetic fluid’s structural parameters encompass the average radius and numerical concentration of MNPs, polydispersity or particle size distribution, interparticle interactions, the formation of clusters and aggregates, the size of the stabilizing shell, and the density and viscosity of the carrier fluid. All these parameters influence the physical properties of magnetic fluid, including magnetization, initial magnetic susceptibility, surface tension, and viscosity, as well as the dynamics of change in the physical properties of magnetic fluid and multiphase systems based on external influences. Let us delve into a more detailed examination of the structural parameters and macroscopic properties of magneto-fluidic systems.

### 4.1. Polydispersity and Interparticle Interactions

As mentioned earlier, researchers initially obtained the first magnetic fluid via grinding magnetite particles in ball mills. The first classical works [69] introduced the description of the size distribution of particles obtained through mechanical grinding. These studies revealed that the distribution of particles derived from mechanical grinding lacks symmetry and cannot be accurately described by a Gaussian distribution. Instead, a lognormal distribution is suggested for interpreting such systems:

(1) $$f\left(x\right)=\frac{1}{xS\sqrt{2\pi }}\exp\left(-\frac{\ln^{2}\left(x/x_{0}\right)}{2S^{2}}\right),$$

where $f\left(x\right)$ is the probability density, x is the diameter of the magnetic core, and $x_{0}$, S are the distribution parameters determined by the results of the experiment.

However, as demonstrated in [70,71], the lognormal distribution inaccurately characterizes the region of large particles, overestimating their actual proportion. A more accurate description of the magnetic fluid can be achieved by employing a two-parameter gamma distribution as follows:

(2) $$f(x)=\frac{x^{\alpha }\exp\left(-\frac{x}{x_{0}}\right)}{x_{0}^{\alpha +1}\Gamma (\alpha +1)},$$

where Γ(α+1) is the gamma function; $x_{0}$, α represent the distribution parameters.

This type of distribution can be used in the future to build theoretical models and analyze experimental data.

Particle size distribution constitutes a crucial factor influencing the dynamics of magnetic fluids under external forces. The theoretical description of a polydisperse system necessitates a fundamentally different approach compared to the ideal monodisperse case. When applying an external magnetic field to a magnetic fluid sample, larger particles easily align along the magnetic field, while the orientation of smaller particles occurs at magnetic field strength values close to saturation. Therefore, polydispersity significantly influences the stability of magnetorheological, magnetic, and dynamic properties of magnetic fluid [72].

Another distinctive feature of magnetic fluid is the presence of interparticle magnetic interactions [73]. For two uniformly magnetized spherical MNPs with magnetic moments $m_{*1}$, $m_{*2}$, the magnetic interaction energy $U_{dd}$ [30] is as follows:

(3) $$U_{dd}\left(r,m_{*1},m_{*2}\right)=-\frac{\mu_{0}}{4\pi }\frac{3\left(m_{*1}\cdot r\right)\left(m_{*2}\cdot r\right)-r^{2}\left(m_{*1}\cdot m_{*2}\right)}{r^{5}},$$

where r is the radius vector between the centers of the particles.

When magnetic moments are oriented in the ‘head-tail’ configuration and magnetic nanoparticles are in close contact, the system attains a minimum energy state, representing the most advantageous position. For evaluating the magneto–dipole interaction of particles in this scenario, an interaction parameter is introduced:

(4) $$\lambda =\frac{\mu_{0}}{4\pi }\frac{m_{*}^{2}}{d^{3}k_{0}T},$$

where d and $m_{*}$ are the diameter and magnetic moment of two identical closely spaced magnetic nanoparticles.

It illustrates the interaction between magnet–dipole interactions and thermal fluctuations. In addition to magnetic attraction forces and thermal fluctuations, forces such as the Van der Waals attraction, steric repulsion, and osmotic forces, contributing to a wedging pressure, affect the interaction between particles [11]. When the parameter significantly exceeds one, the spontaneous self-organization of magnetic nanoparticles occurs, leading to the formation of clusters and aggregates in the shape of rings, chains, or a grid of MNPs. This phenomenon is confirmed by experimental [74,75,76], theoretical work, and computer simulation [77,78]. A schematic representation of the phase space (where the interaction parameter ***λ*** represents the concentration of the solid phase ***φ***) is proposed in [79]. The highlighted areas include the following: I—Langevin superparamagnetic gas; II—interactions between MNPs can be considered using the modified effective field theory; III—the region of chain aggregate formation; IV—the area of closed chain aggregate (rings) formation; V—the area of defective structure formation; VI—the presumed area of percolating grid formation; VII—the area of unknown microstructural formations. Experimental data are available only for regions I-IV of this phase diagram. For magnetic fluids based on the magnetite, stabilized with oleic acid, and with a carrier liquid based on hydrocarbon or oil, the average value of the parameter ***λ*** does not exceed one. Stable aggregates are nearly impossible in such a magnetic fluid. However, under the influence of an external magnetic field, structures can form and disintegrate when turned off. This type of magnetic fluid, associated with areas I and II, is the focus of this paper. This study’s results on the magnetic fluids structure using modern nano-analytical methods are detailed in Section 5.

### 4.2. Features of the Magnetization of Magneto-Fluidic Systems

The first scientific attempts to describe magnetic phenomena date back to the XVIII century [80]. Systematic studies of materials’ responses to a magnetic field began in the XIX century with scientists such as Maxwell, Faraday, Lorentz, and others. Scientists’ research laid the foundation for the classical theory of electromagnetism [81,82,83]. The initial theoretical works that described the process of the magnetization of magnetic fluids [23] considered it within the framework of single non-interacting MNPs. In this scenario, magnetization was estimated using the model of an ideal superparamagnetic gas, which is a model proposed by Langevin [84,85]:

(5) $$M_{L}=nm_{*}L(\xi ),L(\xi )=\mathrm{cth}\xi -\frac{1}{\xi },\xi =\frac{\mu_{0}m_{*}H}{k_{0}T}.$$

This entry of Langevin’s law does not account for the influence of MNPs’ energy in the magnetic field. In certain studies on the Langevin function, the magnetic field strength *H* is substituted with the magnetic induction value *B* [86]. This paper incorporates the consideration of interparticle interactions and the magnetic nature of the magnetic fluid by introducing an effective field. The theoretical approaches to account for the addition of an effective field are discussed below.

The magnetic moments of MNPs align along the field in a process influenced by an external magnetic field. Thermal Brownian fluctuations in the environment impede this process. As the value of the external magnetic field strength increases, the magnetization curve approaches saturation magnetization $M_{S}=nm_{*}$. In this case, when $H>>\frac{k_{0}T}{\mu_{0}m_{*}}$ Equation (5) takes the following form:

(6) $$M=M_{S}-\frac{M_{S}k_{0}T}{\mu_{0}m_{*}H}.$$

The initial magnetic susceptibility for the Langevin magnetization dependence has the following form:

(7) $$\chi_{L}=\frac{\mu_{0}M_{S}m_{*}^{2}}{3k_{0}T}.$$

The magnetization of a polydisperse magnetic fluid is defined as the sum of the contributions of each fraction of magnetic nanoparticles

(8) $$M_{L}\left(H\right)=\sum_{i}m_{*i}n_{i}L(\xi )=n\int_{0}^{\infty }m_{*}(x)f(x)L(\xi )dx,$$

where $m_{*}(x)$ is the distribution function of the magnetic moment of MNPs in the magnetic fluid.

However, Langevin’s law does not account for the dipole–dipole interparticle interactions present in real magnetic fluid.

The first attempt to consider interparticle interactions in the magnetic fluid was made in the effective field model [87]. This model is analogous to the well-known Weiss model [88]:

(9) $$H_{ef}=H+kM,\;\chi =\frac{\chi_{L}}{1-k\chi_{L}},$$

where k is a constant. It establishes the degree of impact of interparticle interactions on Langevin’s susceptibility. The constant $k=\frac{4\pi }{3}$ is equal to *K* when applying the theory of the local Lorentz field. A drawback to this approach is that with an increase in concentration or a decrease in temperature, the equality $\chi_{L}=\frac{1}{k}$ and a second-order phase transition from a paramagnet to a ferromagnet may occur. In the majority of experimental works, such as [89,90], the spontaneous magnetization of a magnetic fluid is not observed.

The Onsager model is the next in terms of approximation. It assumes that the cavity formed by MNPs influences the orientation of the dipoles of neighboring MNPs:

(10) $$\chi =\frac{3\chi_{L}\left(1+\chi \right)}{3+2\chi }.$$

Experimental studies [91] indicate that the Onsager model provides better agreement with experimental data than the Weiss effective field model.

The process of the magnetization of the magnetic fluid was theoretically developed in the medium-spherical approximation of Wertheim [92]. Morozov [93] upgraded the Wertheim approximation for polydisperse magnetic fluid, demonstrating good alignment with experimental data. However, this model requires a substantial number of calculations. The modified effective field theory (MMF) developed by Pshenichnikov lacks this drawback [71]. Within the framework of this theory, it assumes that an effective field, proportional to the Langevin magnetization, additionally acts on MNPs.

An alternative option for considering interparticle interactions in magnetic fluid is the use of the thermodynamic perturbation theory. Within this approach, Ivanov proposed a modified first-order mean field theory (MMF1) [39,94]. It considers the first-order coefficient when decomposed into a series of degrees of freedom. He received clarification in the form of accounting for demagnetizing fields. The refinement is presented in the modified second-order mean field theory (MMF2) [40,95]. Within the framework of this theory, a system of equations describes the equilibrium magnetization of a polydisperse magnetic fluid:

(11) $$M\left(H\right)=n\int_{0}^{\infty }m_{*}\left(x\right)L\left(\xi_{e}\right)f\left(x\right)dx,\;\;\;\;\;\xi_{e}=\frac{\mu_{0}m_{*}\left(x\right)}{k_{0}T}\left(H+\frac{M_{L}\left(H\right)}{3}+\frac{1}{144}M_{L}\left(H\right)\frac{dM_{L}}{dH}\right),\;\chi =\chi_{L}\left[\chi_{L}+\frac{\chi_{L}}{3}+\frac{\chi_{L}^{2}}{144}\right].$$

The MMF2 theory accurately depicts the process of magnetization in polydisperse magnetic fluid with varying concentrations of the magnetic phase across a wide temperature range. This accuracy is substantiated through comparisons with experimental data and computer modeling [40,90,96,97]. An exception to this is the occurrence of abnormally high values of magnetic susceptibility observed at low temperatures in computer simulations [98,99,100].

The concentration of real magnetic fluids can range from fractions of a percentage to concentrated samples with a solid fraction of 20–30%. Researchers tested the MMF2 theory on a broad spectrum of samples with magnetization ranging from 5.0 kA/m to 57.0 kA/m [101]. These theoretical results align with Monte Carlo and molecular dynamics simulation data, as well as experimental magnetization data. This leads to the conclusion that the MMF2 model is versatile and applicable to both diluted and concentrated magnetic fluids.

### 4.3. Relaxation of Magnetic Moments of Nanoparticles in a Magnetic Fluid

**Figure 5:**
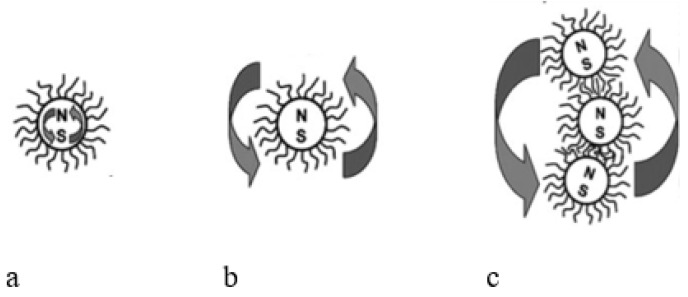
The diagram of the orientation mechanisms of magnetic nanoparticles in a magnetic fluid: (**a**) Néel mechanism of rotation of the magnetic moment inside the particle; (**b**) Brown rotation of an individual particle; (**c**) Brown rotation of the aggregate. Adapted with permission from Ref. [31]. 2014, Joseph, A.; Mathew, S.

The relaxation of magnetization in magnetic fluid is a crucial aspect of MNPs; behavior [102]. When applying an external magnetic field to the magnetic fluid sample, the rotation of the magnetic moment of nanoparticles occurs due to the following two mechanisms of magnetic relaxation: Brown and Néel [103,104,105]. Figure 5 illustrates these mechanisms schematically.

The Brown relaxation mechanism [106] explains the rotational motion of a magnetic nanoparticle in a carrier fluid, with the magnetic moment being fixed along the crystallographic axes. The relaxation time for this mechanism is as follows:

(12) $$\tau_{B}=\frac{3\tilde{V}\eta }{k_{0}T},$$

where $\tilde{V}=\pi \frac{{\left(d+2s\right)}^{3}}{6}$ is the hydrodynamic volume of the particle, including the surfactant, d is the diameter of the particle, *s* is the thickness of the surfactant layer, and η is the viscosity of the carrier fluid.

Large particles exhibit a characteristic feature where thermal fluctuations are suppressed by the energy of magnetic anisotropy. For small nanoparticles, the Néel relaxation mechanism is more characteristic [107]. The Néel relaxation mechanism blocks the rotation of the particle, allowing only the magnetic moment inside the Minds to rotate.

(13) $$\tau_{N}=\tau_{0}\exp\left(\frac{KV}{k_{0}T}\right),$$

where K is the internal anisotropy constant of MNPs, V is its magnetic volume, and the constant is $\tau_{0}$~$10^{-9}$ seconds.

Specific MNPs are characterized by a mechanism with the shortest relaxation time at $\tau_{N}$ or $\tau_{B}$ [25]. This relaxation time is estimated by the following expression:

(14) $$\tau_{eff}=\frac{\tau_{N}\tau_{B}}{\left(\tau_{N}+\tau_{B}\right)}.$$

Both mechanisms, with different relaxation times, depend on the size of MNPs. The Néel mechanism prevails for small particles, while the Brown mechanism prevails for large particles. The critical size for the transition from Néel relaxation to Brown relaxation in magnetite nanoparticles is 13 nm [102]. Past papers, such as [104,108], describe the combination of Brown and Néel MNP relaxation in magnetic fluid.

The relaxation time establishes a connection between the magnetic and rheological parameters. Most experimental studies on magneto-fluidic systems typically employ variable magnetizing external fields to investigate the magnetic response of the sample. Meanwhile, the mechanisms of Néel and Brown relaxation are observed, wherein MNPs deviate from their initial positions while the carrier fluid remains stationary [104,105]. Experimentally performing studies on the viscoelastic properties of surfactants and the transfer of rotational momentum from MNPs to a viscous medium (carrier liquid) presents challenges [108]. It becomes feasible to investigate the relaxation process under external acoustic influences. In this approach, sound is introduced into the magnetic fluid, and elastic vibrations propagating through the carrier fluid impact the surfactant. Surfactant molecules exhibit a certain elasticity and convey perturbations from MNPs to the medium. Conducting the experiment in this manner enabled us to acquire new data on the structure of the magnetic fluid and the mechanism of transmitting elastic influences from the carrier fluid to the MNPs, including its frequency dependence.

### 4.4. Magnetoviscous Effects

Introducing colloidal particles into the carrier liquid increases its effective viscosity. The Einstein formula is applicable for small concentrations of particles, while the Vend formula, which considers hydrodynamic interactions, is used to describe concentrated solutions.

A characteristic of the magnetic fluid is the change in viscosity based on the magnitude of the external magnetic field. We estimated the magnitude of the magnetic fluid response to an external magnetic field using the magnetoviscous effect, defined as follows [59]:

(15) $$MVE=\frac{\eta_{H}-\eta_{0}}{\eta_{0}},$$

where $\eta_{H}$ and $\eta_{0}$ are the viscosity of the magnetic fluid at a given external magnetic field and at a zero magnetic field, respectively.

This dimensionless quantity quantifies the relative increment of viscosity. Applying a magnetic field of a certain magnitude can achieve a relative increase in viscosity at a fixed shear rate.

There is a moment where forces act on a magnetic nanoparticle in a magnetic fluid in a shear flow. When the particles experience this moment, they rotate in a flow with an axis of rotation parallel to the vorticity of the flow. The application of an external magnetic field to the magnetic fluid sample results in the reorientation of the magnetic moment of the MNPs. If the magnetic moment is parallel to the vorticity of the flow, it has no effect on viscosity. However, if the direction of vorticity is perpendicular to the magnetic field, the fixed magnetic moment in the MNPs hinders free rotation. This causes local gradients in the velocity of the carrier fluid near the MNPs, leading to an increase in the viscosity of the magnetic fluid.

Studies on rotational viscosity and the impact of increasing the viscosity of magnetic fluid in a magnetic field were initially described in experimental [109,110] and theoretical [25,111,112] papers. The saturation of the rotational viscosity takes place when MNPs rigidly orient in the magnetic fluid due to the imposition of an external magnetic field.

In works [25,111], a variant of estimating the magnetoviscous effect for a monoparticle dilute magnetic fluid in the case of flow through a circular capillary is proposed:

(16) $$\Delta \eta =\frac{3}{4}\varphi \eta ,$$

where *φ* is the hydrodynamic concentration of magnetic fluid.

In this model, we consider only the Brownian relaxation of the MNP. As demonstrated earlier, real magnetic fluid exhibits the distribution of MNPs by size. Néel and Brown relaxation mechanisms are characteristic of particles of different sizes. In [112,113], the magnetoviscous effect is analyzed with a two-stage mechanism for the relaxation of the magnetization of the magnetic fluid.

The first experimental work demonstrated a twofold increase in the viscosity increment of the magnetic fluid [109] with both longitudinal and transverse orientations of the magnetic field. In [114], the increment in viscosity at the Poiseuille flow in a capillary viscometer under strong magnetic fields for a magnetite-based magnetic fluid sample (volume concentration 0.19–0.24) was 5–6%. A more significant increase in viscosity when applying a magnetic field has been shown for magnetorheological suspensions [115] containing micron-sized magnetic particles. In such systems, the viscosity increased by an order of magnitude [116]. However, they did not exhibit colloidal stability, and phase stratification was observed over time.

A new round of interest in studying the magnetoviscous effect in stable magnetic fluid arose due to the experimental work of Odenbach [117,118]. They investigate the viscosity increment in concentrated magnetic fluid at high shear rates. Experimentally, they observed an abnormal increase in viscosity with an increase in the magnetic field strength. Anomalous viscosity increments significantly exceeded theoretical estimates and could be explained through dipole–dipole interactions and the formation of structures and aggregates in the magnetic fluid. The formation of chain structures has the greatest influence on the magnetoviscous effect, as shown in a series of works by Zubarev [119,120,121]. This team conducted many studies to increase the magnetoviscous effect by manipulating the structure of the magnetic fluid and its physical and chemical properties [122,123,124,125,126].

The influence of the size of magnetic nanoparticles on the magnetoviscous effect was established in [127,128]. They demonstrate that particles with a diameter of less than 10 nm exhibit an insignificant magnetoviscous effect. The impact of MNP concentration in the magnetic fluid is discussed in [129]. It was demonstrated in [129] that an increase in particle concentration results in a heightened interaction between them, leading to a sudden increase in viscosity [130,131]. Another structural parameter influencing the magnetoviscous effect is the size distribution of MNPs [132]. Several studies have explored the impact of surfactant types on the magnetoviscous effect [133,134]. In [135,136], bidisperse systems based on magnetic fluid with the addition of micron particles were examined. The viscosity increase reached 92% of the viscosity of the non-magnetized magnetic fluid.

The intensity and orientation of the external magnetic field impacts viscosity increments in the magnetic fluid. In [126,137], it is demonstrated that the viscosity of the magnetic fluid increases with a change in the magnetic field strength from 0 to 650 kA/m at a constant shear rate. The nature of the change in the magnetoviscous effect is not monotonous. The effect increases sharply up to 100 kA/m and then reaches saturation when approaching a magnetic field of 650 kA/m. In [138], the influence of the rate of increase in the magnetic field on the magnetoviscous effect is considered. It is demonstrated that a slow change in the magnetic field results in a higher viscosity value compared to a rapid increase in the intensity of the external magnetic field. This phenomenon is explained by the formation and destruction of chain structures [139]. One of the ways to describe the magnetoviscous effect is through the use of the Mason number [140,141,142]:

(17) $$M_{N}=\frac{\eta_{c}\dot{\gamma }}{\mu_{0}\mu M_{P}^{2}},$$

where $\eta_{c}$ is the viscosity of the carrier fluid, $M_{P}$ is the magnetization of the particle, and $\dot{\gamma }$ is the macroscopic shear rate.

This parameter combines important factors such as the magnitude of the external magnetic field, the shear rate, the viscosity of the carrier fluid, and the magnetization of the MNPs. It reflects the balance of viscous and magnetic forces [143].

The dynamics of the magnetic fluid are highly sensitive to the dispersion composition of the magnetic fluid [108] and to the uniformity of the rotating magnetic field or the presence of a free surface in the magnetic fluid [144]. For example, in the presence of a freely rotating spindle in the magnetic fluid, under the influence of an external rotating magnetic field, the liquid can rotate both in the direction of rotation of the magnetic field and the opposite direction [145]. Moreover, depending on the rotation frequency of the field, one can observe a decrease in the viscosity of the magnetic fluid [146]. This observation does not align with a single-particle model involving rotating MNPs [147]. These data indicate that even in classical magnetic fluid with an average effective diameter of ~10 nm, there are complex rheological behaviors and the distribution of magnetic forces, which cannot be explained within the framework of early theories [11,12].

The literature describes Brookfield rotary rheometers as the instrument base for measuring the viscosity of magnetic fluid depending on the magnitude of the external magnetic field. In these rheometers, a non-magnetic shaft, disk, or cone rotates within the studied magnetic fluid [148,149,150,151]. Some studies investigate the change in the magnetoviscous effect in the magnetic fluid using capillary viscometers [152].

However, in most applications (such as shock absorbers, vibration dampers, rod seals, accelerometers, inclinometers, speakers, etc.), the active element, which is a specific volume of magnetic fluid limited by the outer shell, undergoes linear movements or oscillations. Therefore, measurements of the elastic-magnetic parameters of the volumes of magnetic fluid levitating in a magnetic field during linear displacements are of particular interest. Linear displacements result from external static and dynamic influences. As magnetic fluid is fluid, studying its oscillatory movements requires the use of a system of pipes either fully or partially filled with magnetic fluid. For example, researchers place U-shaped tubes in an external magnetic field [153,154,155,156,157,158]. In [153], both experimental and theoretical studies of the elasticity coefficient of the system were conducted. The values from the experiment and theoretical evaluation differed by an order of magnitude, with experimental difficulties and a demagnetizing factor indicated as the reasons for this. In [158], researchers investigated the effect of mechanical vibrations on a cylindrical tube filled with magnetic fluid. They wound measuring coils on the tube, and the system was positioned in an external magnetic field. This study focuses on the amplitude–frequency dependencies of the electromotive force induced in measuring coils due to the vibration of the magnetic fluid cylinder in a magnetic field. The analysis of the maximum magnitude was conducted; however, there were no theoretical estimates.

In works [156,157], researchers studied the viscosity of a concentration series of magnetic fluid samples. This paper introduces an original method to study changes in the viscosity of the magnetic fluid. In this method, the magnetic fluid undergoes oscillatory movements in communicating vessels, with the knees of these vessels wrapped with measuring coils. One end is closed with a piezoelectric element, and through the second, vibrations are induced by pulling out the plug. This study demonstrates that for weakly concentrated samples, the theory and experimental data coincide. However, for magnetic fluid with a high concentration, a discrepancy of an order of magnitude is typical. This discrepancy is attributed to the complexity of accounting for the demagnetizing factor. The proposed method requires disassembling the installation and is inconvenient for organizing a large number of measurements.

The viscosity of the magnetic fluid manifests differently depending on the type of process and the orientation of the magnetic field. Volumetric magnetic viscosity is characteristic of laminar flows in pipes and rotational rotation, while wall viscosity is characteristic of vibrational movements and pulsed effects. Existing methods for measuring the viscosity of magnetic fluid in a magnetic field are, however, limited to rotational or capillary methods. However, these methods do not enable us to estimate the contribution of the oscillatory movements of the magnetic fluid in existing systems. Currently, many studies exist that investigate the oscillations of magnetic fluid in a U-shaped tube in a magnetic field and the influence of the magnetoviscous effect on the nature of these oscillations. However, in such a configuration, difficulties arise when considering demagnetizing fields, and it is not possible to assess the wall’s viscosity.

A promising direction for studying the microrheology of magneto-liquid systems involves investigating fluctuations in the volume of a magnetic fluid with a simple cylindrical configuration levitating in an external magnetic field. This approach allows us to assess the magnetic and rheological properties of a magnetic fluid, as well as the microstructure in the thin wall layer of a magneto-liquid system [158,159,160,161,162].

### 4.5. Sound in Magnetic Fluid

For the first time, researchers considered the effect of a plane sound wave on the magnetic fluid in [148]. In [163], the new material was explored as an alternative to piezoelectric converters. Parsons [164] proposed a theoretical model to determine the speed of sound in magnetic fluid, but this statement was found to be incorrect and was refuted in the work published a year later [165], as well as in subsequent studies [166,167]. Another parameter addressed in scientific papers on the acoustics of magnetic fluid is the absorption coefficient of sound vibrations in magnetic fluid. The first experimental studies on this were conducted in [168]. In [168], the researchers found an abnormally high dependence of the absorption coefficient of ultrasonic vibrations in the magnetic fluid on the direction and magnitude of the external magnetic field. This result arose from the incorrect processing of experimental data. Subsequent articles did not confirm these conclusions. In several other works, researchers considered the influence of temperature, the concentration of MNPs, the frequency of sound vibrations, and the presence of aggregates [169,170,171] on the speed of sound and its absorption coefficient in magnetic fluid. The work of Sokolov [172,173] present the results of studies on magnetic fluid using acoustic spectroscopy in the frequency range from 3 MHz to 50 MHz, establishing the relaxation nature of ultrasound attenuation in MF.

However, the most crucial aspect in terms of ferrohydrodynamics is the impact of the magnetic field on the acoustic parameters of the magnetic fluid. In [174], researchers proposed a new mechanism for the dissipation of ultrasound energy in magnetized magnetic fluid due to the oscillation of two particles (dimers) in a viscous medium. The change in the average distance between them has a relaxation character and depends on the angle formed by the dimer axis and the wave vector of the ultrasonic wave. Experimental results were explained using this mechanism [175].

A significant contribution to the study of the acoustic properties of magnetic fluid was made by the scientific school led by Polunin [176,177,178,179]. In his work [180], he considered the theory of excitation of ultrasonic vibrations based on a cylinder of magnetic fluid. Subsequent experimental studies conducted in collaboration with Ignatenko in [181] demonstrated a coincidence with the theoretical estimates. The conversion of elastic vibrations of a magnetized magnetic fluid into a magnetic field disturbance recorded by measuring coils is termed the acoustic magnetic effect (AME) in magnetic fluid. This effect was first theoretically analyzed in [182]. In [182], researchers proposed an expression to estimate the magnetization disturbance of the magnetic fluid in the AME:

(18) $$\delta M=-\frac{nM_{n}+\gamma_{*}M_{T}+i\omega \tau M_{0}}{1+N_{d}M_{H}+i\omega \tau }\cdot \frac{\partial u}{\partial x}.$$

where δM is the increment of the magnetization of the magnetic fluid as a result of the adiabatic deformation of the magnetic fluid under the influence of a sound wave, $N_{d}$ is the demagnetizing factor, $M_{0}$ is the magnetization of the magnetic fluid in the undisturbed state, the parameters $M_{n}\equiv {\left(\frac{\partial M}{\partial n}\right)}_{0}$, $M_{T}\equiv {\left(\frac{\partial M}{\partial T}\right)}_{0}$, $M_{H}\equiv {\left(\frac{\partial M}{\partial H}\right)}_{0}$ characterize the system in the initial state $\gamma_{*}=qTc^{2}{C_{p}}^{-1}$, $q\equiv -\rho^{-1}\partial \rho /\partial T$ is the temperature coefficient, u is the displacement of the magnetic fluid from the equilibrium position under the influence of sound vibrations, and τ is the relaxation time of magnetization.

Experimental confirmation of AME was obtained in [183,184]. However, in these works, the magnetic field in the region outside the tube with the magnetic fluid was not calculated. This calculation is necessary since the measuring coil is not in the magnetic fluid but outside the tube. The expression for the AME in the magnetic fluid contains the magnetization, which, as shown in Section 4.2, depends on the structural features of the magnetic fluid, namely, on the magnetic moments and the size of the MNPs, as well as the shape of the MNPs’ size distribution curve. This establishes the groundwork for developing a new method to study the structure and macroscopic properties of magnetic fluid using the acoustic method [185,186,187]. The method’s utility stems from its relative simplicity and non-invasive nature.

### 4.6. Stability and Aggregation of Magnetic Particles in a Magnetic Fluid in an Inhomogeneous Magnetic Field

The stratification of magnetic fluid under the influence of an external inhomogeneous magnetic field is termed magnetophoresis. This process can have a negative effect on the magnetic fluid sample when prolonged exposure to an inhomogeneous magnetic field leads to changes in the physical parameters and stability of the sample [188,189,190] and a positive effect. This positive effect is utilized in microfluidic separation systems [191,192], where the separation of magnetic nanoparticles of different sizes is controlled by an external inhomogeneous magnetic field [193].

Further experimental studies aimed to describe the effect of the deterioration of magneto-fluidic sealants. In these studies, researchers fill the magnetic fluid into a thin gap, and it is positioned in a highly inhomogeneous magnetic field. In works led by Naletova [194,195], they considered magnetophoresis in the magnetic fluid poured into a 150 mm long test tube placed in an inhomogeneous magnetic field. The magnetic field was generated by an electromagnet with conical tips. The samples of magnetic fluid with large particles were used, in which stratification was observed for 6 h. These samples differ from the magnetic fluid used in the industry and are more stable. A collaborative effort between engineers from ‘Ferrolabs Inc.’ (Dulles, VA, USA) and a team led by Bashtovy is dedicated to studying commercial magnetic fluid [7,196]. In [7,196], they examined the processes of magnetophoresis in sealers and shock absorbers.

The theoretical descriptions of magneto-diffusion and magnetophoresis processes are discussed in [197,198,199]. The equations derived in [197,198,199] vary in the completeness of accounting for the effects of magnetophoresis, gradient diffusion, sedimentation, the anisotropy of transfer coefficients, and interparticle interactions. In [188], a magneto-diffusion equation was proposed based on the decomposition of the free energy of a system of interacting dipoles into a series in terms of density. This equation describes the spatial and temporal changes in the volume fraction of single-domain superparamagnetic nanoparticles in a concentrated magnetic fluid. As demonstrated in [189,200], this equation adequately portrays the temporal and spatial distribution of superparamagnetic nanoparticles in concentrated magnetic fluid in an inhomogeneous magnetic field, provided there are no aggregates and large particles present.

As mentioned above, magnetic fluid is classified as an optically opaque liquid. An examination of the magnetic fluid for the lumen is only possible in a thin layer poured into a transparent cell. The thickness of the thin layer varies from tens of microns for concentrated magnetic fluids to about 1 mm for low-concentrated magnetic fluids. Therefore, as a rule, light rays are not utilized to study optical effects, such as the rotation of the plane of polarization, scattering, and absorption of light in layers with a thickness of ~1 mm. Studies based on optical methods addressing stability in thin layers of magnetic fluid in the presence of the aggregation of magnetic particles are presented in [189,200].

In the works [189,200], researchers employed a photometric method to study the dynamics of MNPs in magnetic fluid under the influence of an inhomogeneous magnetic field. In the photometric method, a laser generated the luminous flux in the red region of the spectrum, with the thickness of the magnetic fluid layer ranging from 0.02 mm to 0.2 mm. The processes of magnetophoresis and diffusion of magnetic nanoparticles in a magnetic fluid layer under the action of a gradient field (Δ*H*/Δ*x* = 3 × 10^6^ A/м^2^) are described. In the experiments, the time interval required to establish concentration equilibrium was estimated. For dilute magnetic fluid (volume concentration of magnetite particles from 2% to 5%), the time to establish an equilibrium concentration distribution along the cell length of 2 mm was ~12 days.

A renewed surge of interest in studying the process of magnetophoresis in magnetic fluid has arisen due to the development of microfluidic technologies [191,193]. The inhomogeneous magnetic field enables an increase in the concentration of MNPs and the controlled capture and transport of MNPs under the influence of externally controlled and space-movable magnetic field sources. In this case, various surfactants capable of combining with various biological objects can be used to functionalize the surface of the particles [201]. These include cancer cells [46], DNA [202], antibodies [203].

Based on the information presented in this paragraph, it is evident that a considerable amount of work has been dedicated to studying magnetophoresis and magnetodiffusion processes. However, the issues regarding the flow of these processes in recently developed axial magnetic systems, based on assemblies of annular unipolar magnets [204,205], that are capable of creating magnetic field with a strength of 500–2000 kA/m in areas of a few centimeters, as well as in the magnetic field created by annular permanent magnets (RPM), the geometry and configuration of which allow a test tube to be placed with a separable magneto-fluidic system into the hole of the magnet, and then increasing the concentration of MNPs in a certain area by moving the test tube, remain underexplored. Examining the processes of magnetophoresis and magnetodiffusion in the magnetic fields of the aforementioned sources enabled us to investigate the dynamics of MNPs in magneto-fluidic systems under conditions of prolonged exposure to extremely high values of a magnetic field strength and gradient. These conditions often occur in the gaps of magnetic-liquid seals, sensors, and microfluidic systems containing magnetic fluids.

## 5. Experimental Methods for Studying the Structure, Properties, and Dynamics of Magneto-Fluidic Systems

As magnetic fluids contain magnetic nanoparticles, measuring the size of the latter directly is only possible using nanoanalytical equipment. Historically, transmission electron microscopy was the first method used to study MNPs [206,207]. Subsequently, researchers conducted direct size measurements of MNPs in the magnetic fluid using scanning electron microscopy [208,209], atomic force microscopy [210,211], and magneto-force microscopy [212].

**Figure 6:**
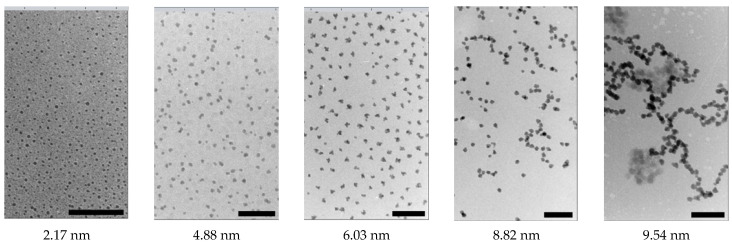
Cryo transmission electron microscopy images of magnetic fluid samples with different particle sizes. Adapted with permission from Ref. [213]. 2003, Butter, K.; Bomans, P.H.H.; Frederik, P.M.; Vroege, G.J.; Philipse, A.P.

The transmission electron microscopy technique is sensitive only to the magnetic core of particles. The light elements of the organic shell do not provide sufficient contrast. To conduct a study using transmission electron microscopy, researchers expose samples of magnetic fluid to drying. However, this process blocks kinetic properties in the replica. A more advanced method is the study of samples frozen at low temperatures (cryo transmission electron microscopy), with which the team led by Philipse managed to record the formation of linear dipole structures in situ in the magnetic fluid in a zero magnetic field [213,214]. In these works, researchers recorded the transition from individual particles at a size of 2.17 nm to randomly oriented linear aggregates and branched chains at a size of 9.54 nm. The microscopic images of these structures are shown in Figure 6.

In [213,214], researchers studied magnetic fluid with iron particles, which exhibit a very strong dipole–dipole interaction. The formation of loosely coupled clusters (MNP clouds) is possible in magnetite magnetic fluid [215,216].

Some publications consider studies of magnetic fluid using scanning probe microscopy, namely atomic force microscopy [210,211] and magneto-force microscopy [212]. Their result was a three-dimensional surface reflecting the topology of the substrate on which the deposition of MNP magnetic fluid was carried out. The tip of the cantilever repeats the profile of deposited MNPs coated with surfactants. Sample preparation also plays a special role here, involving obtaining a monolayer of nanoparticles, as well as taking into account the interaction of the tip of the cantilever and surfactant, the molecule of which can stick to the tip, leading to the distortion of the measurement results.

Another method for studying the structure of magnetic fluid is small-angle X-ray scattering [217,218]. It is sensitive to the size of the magnetic core of the magnetic fluid nanoparticles. Additionally, small-angle neutron scattering [219,220,221] is among the methods used to study the structure of magnetic fluid. These unique methods extract information from the diffraction pattern of X-ray scattering or the neutron flux at small scattering angles, enabling the study of structures of objects in the nanometer range (1–100 nm). Studying the interaction of magnetic nanoparticles poses a challenging problem. Traditional approaches based on concepts such as dipole–dipole, Van der Waals, and other types of interactions [11,12] appear to be insufficient. To explain this effect, the authors invoke the so-called depletion attraction [222]. This example illustrates that, during the interaction of MNPs in the magnetic fluid, it is necessary to consider the short-acting forces between the core of the magnetic particle, the surfactant, and the dispersed medium. At the same time, the interaction between the MNPs’ core and the surfactant occurs during the synthesis of magnetic fluid. For instance, surfactants are physically adsorbed onto the MNPs’ nuclei in aqueous media [12]. In hydrocarbon liquids, a mixture of magnetite particles and oleic acid, when heated, can undergo chemical adsorption, resulting in the formation of iron oleate.

Another cost-effective method for studying the structure of magneto-fluidic systems involves interpreting experimental dependencies of the physical properties of magnetic fluid under various external influences. The most common approach is magnetogranulometric analysis, which relies on measuring the magnetization curve of a magnetic fluid [206]. Measuring devices such as vibration magnetometers [223], magnetometers based on a superconducting quantum interferometer [224,225,226], and the magnetization measurement of the magnetic fluid using the ballistic method or mutual inductance bridges [71] are employed. The magnetogranulometric analysis technique also determines the diameter of the magnetic core of the nanoparticle without considering the presence of a thin non-magnetic layer on the surface of the MNPs, as well as surfactants.

## 6. Controlled Active Magneto-Fluidic Systems

One of the most common applications of magneto-fluidic systems is their ability to externally control the physical parameters and dynamics of such systems using external magnetic fields [227,228]. Magnetic particles in such systems are modified with specific and challenging-to-synthesize surfactants. Surfactants can interact and bind only with certain types of biological objects (cells, proteins, viruses, etc.) or organic compounds [229]. In the future, these systems can be sorted using magnetic parameters or transported to a specific area (magnetically controlled delivery).

An alternative option for using extremely complex surfactants in synthesis is the direct (label-free) manipulation with external magnetic fields of non-magnetic objects (gas bubbles, cavities, drops of non-magnetic liquid, non-magnetic solid inclusions, biological inclusions, and organic compounds) dispersed in magnetic fluid [44,192,230,231]. To implement this approach, firstly, biocompatible magneto-fluidic systems are needed, as well as sources of inhomogeneous magnetic fields. These sources are controlled by adjusting the magnitude of the magnetic field strength using current sources connected to electromagnets with cores of various configurations and controlled movement in the space of magnetic field sources using a variety of mechatronic systems.

Many works have been published over the past few years, describing a new topical method of influencing magneto-fluidic systems using spatially inhomogeneous magnetic fields. The greatest interest is caused by magnetic systems that allow the creation of magnetic fields of a special configuration. These systems have a region of zero magnetic field strength (the “magnetic vacuum” region). In such fields, the magnetic field gradient changes its direction [232,233]. In the future, this area of the magnetic field will be designated as the “magnetic vacuum” region. However, in the scientific and technical literature, there are examples of other terminology related to magnetic fields created by an annular permanent magnet. For example, “special points of the magnetic field of an annular permanent magnet”, “magnetic potential pits”, “points of magnetic anomalies”, etc. Therefore, the chosen term is not the only possible one.

The magnetic vacuum region allows for controlled dynamics of non-magnetic inclusions in the magnetic fluid under the influence of an inhomogeneous external magnetic field. Recent works [234] describe assemblies of permanent magnets in which the “magnetic vacuum” region has a different configuration. But the most common of such systems are ring permanent magnets [235]. They can be used as sources of inhomogeneous magnetic fields in this work.

### 6.1. Dynamics of Non-Magnetic Gas and Liquid Inclusions in a Magnetic Fluid

Studies of the dynamics of gas inclusions, such as cavities and bubbles in the magnetic fluid, are of scientific interest due to the emerging possibility of controlling mass transfer processes using a controlled external magnetic field. Visualizing the behavior of a non-magnetic liquid and gas phase poses a challenge because magnetic fluids are opaque. In previous studies, X-rays [236,237], ultrasonic flow detection [238], and infrared radiation [239] were employed to visualize the behavior of the magnetic fluid. At the same time, recording the disturbance of the magnetic field caused by the movement and pulsation of a non-magnetic bubble could be achieved using a measuring coil surrounding a vessel with magnetized magnetic fluid [239]. The obtained data can be utilized to study the dynamics of non-magnetic inclusions.

Many experimental studies focus on the shape, velocity of bubbles, and decay of gas jets in the magnetic fluid under external magnetic fields. In [238], an experiment was conducted to determine the effect of an inhomogeneous magnetic field on the dynamics of bubbles in the magnetic fluid. The magnetic field was generated by an annular electromagnet. It was established that the proportion of gas inclusions in a two-phase magneto-liquid flow decreases with an increase in the magnetic field. The deformation and translational motion of a gas bubble in a fluid-filled transparent thin channel were considered. Experimental data show that the movement of the gas bubble slowed down in the region of the positive gradient of the magnetic field. Conversely, in the region of the negative gradient of the magnetic field, the movement accelerated while the bubble lengthened in the direction of the magnetic field.

The direct visualization of the dynamics of the gas phase in the magnetic fluid is possible in a thin slit or transparent cell. In article [240], researchers studied the influence of a homogeneous magnetic field on the dynamics of droplets and bubbles in the magnetic fluid. The article also considers the influence of the dipole–dipole interaction between droplets and bubbles on the instability of such flows, determining the critical values of the flow parameters and magnetic field strength. Paper [241] investigated the shape of bubbles in magnetic fluid and several non-magnetic liquids (water, sucrose solution with different concentrations, and an aqueous solution of tetramethylammonium hydroxide). The experiments were conducted in two versions of flat transparent channels with thicknesses of 1 and 2 mm. This study revealed that the shape of the bubble in the magnetic fluid changed due to the diffusion of surfactants on the bubble interface. Studies of non-magnetic liquid and gas inclusions are presented in [242,243], where the dynamics of bubbles and droplets in a flat slit are examined.

In [244], the study examines the dynamic behavior of a gas bubble in a magnetic fluid subjected to a homogeneous magnetic field. The equation proposed aims to estimate the size of a gas bubble based on the balance of forces.

In [245,246], researchers conducted studies on gas bubbles floating in a tube filled with a magnetic fluid. Measuring coils wound around the tube recorded the induced electromotive force (EMF) resulting from the disturbance of the magnetic field via a non-magnetic bubble in the magnetic fluid. The experiment led to the proposal of a bubble counter-recorder. In the majority of the articles mentioned above, multiphase systems based on magnetic fluid were subjected to the influence of a uniform magnetic field. Only [238] provides a study on the influence of an inhomogeneous magnetic field on the dynamics of gas inclusions in the magnetic fluid. This investigation explores the velocity and deformation of gas bubbles in the magnetic fluid subjected to an inhomogeneous magnetic field created by an annular electromagnet. It has been demonstrated that the size of bubbles floating in a magnetic field depends on the parameters of the magnetic field, as well as on the rate of gas supply to the capillary. The latter imposes certain restrictions on the use of such a system as a gas dispenser in conditions of changing flow.

A theoretical model describing the dynamics of the motion and oscillation of the gas bubble wall in a magnetic fluid under the simultaneous influence of an external magnetic field and an acoustic signal is presented in [247]. This model introduces a theoretical framework for nonlinear motion. In [247], X-ray phase-contrast imaging methods were employed to study the behavior of gas bubbles in the magnetic fluid. This study reveals that the application of a magnetic field resulted in the deformation of bubbles in the magnetic fluid and the formation of linear chains composed of several bubbles. Numerical simulation data lead to the conclusion that the magnetophoretic force is the underlying cause of the aggregation of gas bubbles in the magnetic fluid.

In [248], the study focuses on the process of capturing and flooding an air cavity from the surface of a magnetic fluid. The magnetic fluid is poured into a horizontal cylindrical tube under the influence of an annular permanent magnet. The magnet moves coaxially with the tube. The classical monograph [11] provides the initial report on the possibility of sealing a tube with magnetic fluid using an inhomogeneous magnetic field created by an annular permanent magnet. However, neither [11] nor subsequent scientific articles prior to the publication [248] reported a controlled effect on the air cavity in the magnetic fluid using an incoming magnetic field. As the annular permanent magnet moves downwards, ponderomotive forces from the inhomogeneous magnetic field press the air cavity in the magnetic fluid against the bottom of the tube. When a specific pressure threshold is reached in the magnetic fluid, the air bubble detaches from the cavity and rises. Once outside the magnetic vacuum region, the air bubble undergoes elastic vibrations in the magnetic fluid. These oscillations are accompanied by electromagnetic and acoustic radiation.

The construction of mathematical models for magneto-fluidic systems in a magnetic field is theoretically one of the most complex phenomena in the physics of magnetic fluids. This complexity arises because the magnetic field is distorted at the interface due to the difference in magnetic permeability. Alongside these mentioned difficulties, it is necessary to account for hydrodynamic flows with free boundaries. The most popular methods for the theoretical and computer modeling of the shape of gas bubbles in magnetic fluid, including in external inhomogeneous magnetic fields, involve the use of numerical algorithms to solve Maxwell’s equations, the Young–Laplace equation, and the Navier–Stokes equations [247,249,250,251,252], various modifications of the volume of fluid method [253,254], and modeling based on the Boltzmann lattice [254,255,256,257].

Numerous studies focus on the dynamics of magneto-fluidic systems containing gas and liquid non-magnetic inclusions. Most of these works explore the effects of either homogeneous magnetic fields or the inhomogeneous magnetic fields of simple configurations. Currently, there are several works [258,259,260,261] that investigate magneto-fluidic systems under the influence of inhomogeneous magnetic fields of a special configuration containing a magnetic vacuum region where magnetic levitation is observed. This complexity arises from the experimental challenges posed by the optical opacity of magnetic fluids. Directly observing the dynamics of non-magnetic inclusions in magneto-fluidic systems is possible only in thin, optically transparent channels or measuring cells using high-speed video recording systems. An alternative option for studying the dynamics of non-magnetic inclusions, not considered before, may be the acoustic–magnetic indication of magnetization disturbances caused by moving or oscillating liquid or gaseous non-magnetic inclusions in magneto-fluidic systems located in inhomogeneous magnetic fields [262,263].

### 6.2. Levitation of Non-Magnetic Inclusions in Magnetofluidic Systems

A popular area of research related to magnetism in liquid media is the application of the magneto-Archimedean effect in magnetic levitation (MagLev) [235,264,265,266]. Its essence is based on the fact that when solid or liquid diamagnetic samples are suspended in a magnetic fluid, and the system is in a strong magnetic field, interaction with the latter leads to a force acting against gravity. Moreover, the spatial distribution of the generated force forms a stable trap that allows the manipulation of these samples [13,267,268]. Currently, this effect has found many applications in materials science, physics, physical chemistry, and even biochemistry. These applications include manipulating solid and liquid particles [235,269], high-precision measurements of the density of micro-samples and even living cells [265,267,268,269,270,271], non-contact self-assembly processes [272,273], etc.

The standard modern configuration of the magnetic system used in studies of the MagLev phenomenon in magneto-fluidic systems consists of two coaxial counter-oriented annular permanent magnets, which almost constantly provide a gradient along the axial line of the system [274,275,276]. Accordingly, the working area is around the central point between the magnets, where the magnetic field strength magnitude crosses the zero value. At the same time, some recent studies have involved systems with a single permanent magnet in the form of a ring or a rod of a special shape [277,278,279].

This form of source gives rise to the topology of the magnetic field, which has a more complex shape compared to the traditional system of two counter-directed magnets. Choosing the appropriate size and aspect ratio of a permanent magnet leads to the realization of a zero magnetic field and an almost constant gradient of the magnetic field in its vicinity, as well as the inversion of the gradient. The latter function can be used to capture and separate a mixture of suspended samples, where some have a density less than that of a paramagnetic fluid carrier [236,278]. Therefore, it is necessary to obtain the spatial distribution of the magnetic field for such MagLev systems. In principle, the corresponding calculations do not require complex techniques [280]. Calculations can be performed using various standard scientific programs, representing a generally accepted approach. Simultaneously, to ensure the accurate calibration of real installations, it may be necessary to verify the actual configuration of the magnetic field, especially in areas distant from the axis of the system (this is also desirable for conventional structures, particularly when operating in a wide three-dimensional space [267,275,276]).

Conventional magnetometers produce a discrete set of values measured at points, making the interpolation of data points unavoidable. Consequently, the finite dimensions of the sensitive elements of magnetometers can lead to inaccuracies, which can be significant. This is explained by the fact that even when modeling the distribution of the magnetic field using software, some experimentally measured values are used to calibrate the input parameters of the magnetization of the magnet. These finite-size effects prevent measurements in miniature magnetic levitation systems [278]. A new method for scanning the topology of the magnetic field, as proposed in [280,281], aims for a more accurate assessment of the size of the magnetic field region for magneto-fluidic systems with different parameters, predicting the behavior of non-magnetic inclusions in such systems under the influence of inhomogeneous magnetic fields.

## 7. Conclusions

Analyzing experimental and theoretical works dedicated to magnetic fluids and systems based on them, which pertain to the study of their structure, macroscopic properties, and dynamic behavior under external influences, reveals that this topic holds scientific interest. Research in this field is published in leading journals with a high impact factor and is reviewed at prominent specialized conferences. These studies have given rise to a new multidisciplinary scientific direction as follows: the physics of magnetic soft materials (soft magnetic matter physics). It encompasses tasks primarily in condensed matter physics and magnetism, as well as magnetic hydrodynamics, inorganic, organic, and colloidal chemistry, computational and computer modeling, acoustics, engineering, and applied sciences. Addressing scientific challenges related to the investigation of the structure, macroscopic properties, and dynamics of behavior under external influences on magnetic fluid and the systems based on them can be beneficial in creating and predicting the behavior of new multiphase “smart” materials in various application conditions.

As demonstrated in this review, numerous works focus on the effects of various physical fields (magnetic, acoustic, electrical, hydrostatic, and hydrodynamic) on magneto-fluidic systems. Interest in researching this topic has been growing in recent years, driven by the development of microfluidics, micro-separation, and controlled self-assembly and transport in colloidal systems. However, despite this, many issues remain unexplored, and some aspects are not fully understood.

Nanoanalytical methods, including transmission electron microscopy, scanning electron microscopy, atomic force microscopy, and magneto-force microscopy, are employed in a static mode for studies, except for cryo transmission electron microscopy, which examines the hardened replicas of magnetic fluids. Simultaneously, noninvasive, acoustic, magnetic, and mechanical effects on magneto-fluidic systems result in various dynamic responses, offering new insights into the structure and properties of these systems. Acoustic waves, when acting on MNPs coated with surfactants, can provide information about hydrodynamic size in contrast to known methods such as magnetogranulometric analysis, which estimates the diameter of the magnetic core. This is different from microscopic and X-ray research methods, where replicas are examined. Existing models of the acoustomagnetic effect consider the polydispersity of the magnetic fluid, the magnetoviscous effect, and the relaxation of particles under acoustic influences. Incorporating these parameters enables more accurate predictions of the structure, macroscopic properties, and dynamics of the behavior of the magnetic fluid and systems based on them when studying them using a non-invasive method based on the acoustic–magnetic effect.

In the most common applications of magnetic fluid, such as dampers, sealers, sensors, acoustic systems, and other devices, the volume of magnetic fluid undergoes translational oscillating movements. Studying the changes in the properties of magnetic fluid under such mechanical influences is a prospective problem. Analyzing the alteration in the wall viscosity of the magnetic fluid cylinder under external influences, including the long-term effects of a magnetic field, can provide insights into the structure formation of magnetic fluid used in practical applications.

The investigation of the phenomenon of the magnetic levitation of non-magnetic gaseous, solid, and liquid inclusions in a magnetic fluid under the influence of an inhomogeneous magnetic field in microchannels of various shapes is a pressing topic. Research in this area can contribute to the development of microfluidic dispensers and counters.

The advancement of research in this interdisciplinary direction, the establishment of new experimental facilities, the accumulation of experimental data, and the formulation of adequate theoretical models are crucial scientific challenges. Solving these problems enables the prediction of the dynamics and properties of magneto-fluidic systems, as well as an understanding of the interaction between their macroscopic properties and microstructural states under magnetic, mechanical, and acoustic influences, thereby offering practical applications.

## Figures and Tables

**Table 1:** Physical parameters of magnetite.

the density of massive magnetite $\rho_{f},\;\frac{kg}{m^{3}}$ [61]	5240
cell parameters	*a* = 0.8397 nm, *Z* = 8
curie point $T_{C},\;K$ [62]	858
saturation of magnetization $M_{S},\;kA/m$	490
the constant of crystallographic anisotropy $K,\;J/m^{3}$	$1.1\times 10^{4}$

## Data Availability

Not applicable.
